# Aging anxiety and menopause-specific quality of life: the parallel mediating roles of self-efficacy and self-acceptance

**DOI:** 10.3389/fpubh.2026.1836259

**Published:** 2026-08-03

**Authors:** Jungmi Kang

**Affiliations:** Department of Nursing, Sun Moon University, Asan, Republic of Korea

**Keywords:** aging anxiety, menopause-specific quality of life, parallel mediation, self-acceptance, self-efficacy

## Abstract

**Background:**

Menopause is a significant life transition during which women experience both physical and psychological changes. Aging anxiety has been identified as a key psychological factor that may negatively affect menopause-specific quality of life. However, the mediating roles of self-efficacy and self-acceptance in the relationship between aging anxiety and quality of life have not been sufficiently examined.

**Objective:**

This study aimed to examine the parallel mediating roles of self-efficacy and self-acceptance in the relationship between aging anxiety and menopause-specific quality of life among women experiencing menopausal symptoms.

**Methods:**

This study used secondary data from 114 women experiencing menopausal symptoms (*N* = 114). Aging anxiety was treated as the independent variable, self-efficacy and self-acceptance as mediators, and menopause-specific quality of life (MENQOL) as the dependent variable. A parallel mediation analysis with 5,000 bootstrap samples was conducted.

**Results:**

Aging anxiety showed a significant direct effect on menopause-specific quality of life (B = 1.40, *p* < 0.001). The total indirect effect through self-efficacy and self-acceptance was statistically significant (B = 0.36, 95% CI [0.03, 0.71]). However, the specific indirect effects through self-efficacy and self-acceptance were not statistically significant, as their confidence intervals included zero.

**Conclusion:**

These findings suggest that aging anxiety may influence menopause-specific quality of life both directly and indirectly through the combined effects of self-efficacy and self-acceptance. Self-efficacy and self-acceptance may operate jointly rather than independently as mediators in this relationship. Therefore, interventions aimed at reducing aging anxiety and strengthening psychological resources may be beneficial for improving quality of life among women experiencing menopausal symptoms.

## Introduction

With the increase in life expectancy, women are spending a longer proportion of their lives in midlife and beyond, making the health and quality of life of women experiencing menopausal symptoms an important public health issue (1, 2). Globally, a substantial proportion of middle-aged women experience moderate to severe menopausal symptoms, which are associated with increased healthcare utilization and economic burden, highlighting the magnitude of menopause-related health concerns at the population level (1, 3). Menopause is characterized by a decline in ovarian function and reduced estrogen levels, leading to a wide range of physical and psychological changes, including hot flashes, sleep disturbances, fatigue, depression, and anxiety (3, 4). These changes may negatively affect daily functioning and psychological well-being, ultimately reducing quality of life (5).

The quality of life of women experiencing menopausal symptoms is a multidimensional construct influenced not only by physical symptoms but also by psychological and social factors (2). In particular, menopause-specific quality of life (MENQOL) reflects individuals’ subjective evaluations of changes experienced in physical, psychological, and sexual domains and is considered an important indicator of health status and adaptation during menopause (6, 7). Previous studies have reported that menopausal quality of life is associated with various factors, including health status, psychological characteristics, and social support, which may interact to influence overall well-being (2, 8, 9).

Recently, aging anxiety has received increasing attention as an important psychological factor affecting quality of life among women experiencing menopausal symptoms (10). Aging anxiety refers to the fear and concerns associated with age-related changes, such as physical decline, health problems, and shifts in social roles, as conceptualized by Lasher and Faulkender (11). Negative perceptions of aging may increase psychological stress and reduce life satisfaction and psychological well-being, thereby adversely affecting quality of life (10, 12). Empirical studies have reported that higher levels of aging anxiety are associated with lower quality of life, highlighting its significance in psychological adaptation during menopause (7, 10).

Psychological resources may serve as protective factors that buffer the negative effects of aging-related concerns on quality of life (10, 13). Among these, self-efficacy refers to an individual’s belief in their ability to successfully perform behaviors required to achieve specific outcomes and has been widely recognized as a key determinant of health behavior and psychological adaptation (14). According to Bandura’s social cognitive theory, individuals with higher self-efficacy are more likely to actively cope with stress and engage in health-promoting behaviors (14, 15). Previous studies have reported that higher self-efficacy is associated with greater psychological well-being and improved quality of life among women (16–18).

In addition, self-acceptance, defined as the ability to accept oneself fully, including both strengths and limitations, is a core component of psychological well-being (19). Ryff’s model of psychological well-being suggests that self-acceptance plays a critical role in mental health and life satisfaction (20). For women experiencing menopausal symptoms, who experience both physical changes and shifts in social roles, self-acceptance may function as an important psychological resource that facilitates adaptation and enhances quality of life (21).

Based on Bandura’s social cognitive theory and Ryff’s model of Psychological Well-Being, aging-related anxiety can influence individuals’ cognitive and emotional processes, which in turn affect their adaptation and quality of life (15, 19). Within this theoretical framework, self-efficacy and self-acceptance can be understood as distinct yet complementary psychological resources. Self-efficacy reflects individuals’ beliefs in their ability to cope with challenges and engage in adaptive behaviors (14, 15), whereas self-acceptance refers to an individual’s capacity to maintain a positive and accepting attitude toward oneself despite age-related changes (19, 20). Therefore, these two variables are more likely to operate simultaneously rather than sequentially, supporting their role as parallel mediators in the relationship between aging anxiety and menopause-specific quality of life (14).

Previous studies have reported that self-efficacy is positively associated with psychological well-being and quality of life, particularly among women experiencing menopausal symptoms (16, 17). Similarly, self-acceptance has been identified as an important factor related to life satisfaction and emotional well-being, especially in the context of aging (22, 23). However, most prior studies have examined these psychological factors independently, with limited attention to their combined or simultaneous effects (2, 9) In addition, some studies have reported associations between psychological factors and quality of life, suggesting that these relationships may differ depending on contextual and individual characteristics (2, 9). Therefore, an integrative approach that considers multiple psychological resources simultaneously is needed to better understand their collective influence on menopause-specific quality of life.

Recent studies suggest that the relationship between influencing factors and quality of life may not be explained solely by direct effects but also through underlying psychological mechanisms such as self-efficacy (24, 25). Furthermore, psychological resources, including self-acceptance, have been identified as key factors in explaining the relationship between emotional experiences and quality of life (22). However, previous studies have primarily focused on simple associations between aging anxiety and quality of life, with limited attention to the psychological mechanisms underlying this relationship (10). Although the roles of self-efficacy and self-acceptance have been examined individually, few studies have investigated their simultaneous effects within an integrated framework (22, 24).

Therefore, this study aimed to examine the effect of aging anxiety on menopause-specific quality of life among women experiencing menopausal symptoms and to investigate whether self-efficacy and self-acceptance serve as parallel mediators in this relationship.

Based on the theoretical framework and previous findings, the following hypotheses were formulated:

*H1:* Aging anxiety is significantly associated with menopause-specific quality of life.

*H2:* Self-efficacy mediates the relationship between aging anxiety and menopause-specific quality of life.

*H3:* Self-acceptance mediates the relationship between aging anxiety and menopause-specific quality of life.

By identifying these psychological mechanisms, this study aims to provide a more comprehensive understanding of factors influencing quality of life and to offer foundational evidence for developing interventions to promote psychological adaptation and improve quality of life among women experiencing menopausal symptoms.

## Materials and methods

### Study design

This study was a cross-sectional study based on secondary data analysis using previously collected online survey data. The study was conducted to examine the parallel mediating roles of self-efficacy and self-acceptance in the relationship between aging anxiety and menopause-specific quality of life.

### Participants

The participants of this study were 114 women experiencing menopausal symptoms, and the data were obtained from an existing dataset collected by the researcher through an online survey. Data were collected over a 15-day period from January 1 to January 15, 2025. Participants voluntarily took part in the study after receiving a full explanation of the study purpose and procedures.

Participants were recruited through a study information notice posted on a local online community. The notice included details on the study purpose, procedures, the right to withdraw at any time, privacy protection, and the scope of data use for research purposes, including potential secondary analysis. The survey was designed such that participants could proceed only after reviewing this information and providing informed consent by checking a consent box.

A non-probability convenience sampling method based on voluntary participation was used. A total of 118 participants were initially recruited, considering an anticipated dropout rate of 15%. After excluding four incomplete responses, data from 114 participants were included in the final analysis.

To ensure response accuracy, all survey responses were carefully examined for missing values, incomplete responses, and logically inconsistent answers. Responses that did not meet pre-defined criteria—for example, contradictory answers across items, impossible values, or repetitive identical patterns—were removed prior to analysis.

The required sample size was calculated using G*Power 3.1, with an effect size of 0.15, a significance level of 0.05, statistical power of 0.90, and three predictors, indicating a minimum sample size of 102 participants.

As this study used secondary data, ethical approval was obtained from the Institutional Review Board of Sunmoon University (IRB No. SM-202511-028-1). All data were anonymized and used solely for research purposes.

### Measures

#### Aging anxiety

Aging anxiety was measured using the Aging Anxiety Scale developed by Lee and Yoo (26). The instrument consists of 19 items, each rated on a 5-point Likert scale ranging from 1 (“strongly disagree”) to 5 (“strongly agree”). Total scores range from 19 to 95, with higher scores indicating greater aging anxiety. For the present study, scores were calculated as mean item scores rather than summed total scores to allow interpretation within the original response scale and to facilitate comparison across variables. The original instrument reported a reliability of Cronbach’s *α* = 0.91. In the present study, Cronbach’s α was 0.93.

#### Self-efficacy

Self-efficacy was measured using the Self-Efficacy Scale originally developed by Sherer et al. (27) and later translated into Korean by Hong (28). The instrument consists of 23 items, each rated on a 5-point Likert scale ranging from 1 (“strongly disagree”) to 5 (“strongly agree”). Total scores range from 23 to 115, with higher scores indicating greater self-efficacy. As with other variables, scores were expressed as mean item scores rather than total scores to ensure comparability and ease of interpretation across measures. The original instrument reported Cronbach’s *α* = 0.78, and the Korean version reported Cronbach’s α = 0.77. In the present study, Cronbach’s α was 0.91.

Although this scale was adapted in 1994, it has been widely used in Korean populations and has demonstrated acceptable reliability and validity across multiple studies. In the present study, the scale also showed high internal consistency, supporting its continued use.

#### Self-acceptance

Self-acceptance was measured using the Revised Unconditional Self-Acceptance Questionnaire developed by Chamberlain and Haaga (23) and later translated and validated in Korean by Chu and Lee (29). The instrument consists of 15 items, each rated on a 5-point Likert scale ranging from 1 (“strongly disagree”) to 5 (“strongly agree”). Total scores range from 15 to 75, with higher scores indicating greater self-acceptance. Scores were calculated as mean item scores rather than summed total scores to maintain consistency across instruments. The original instrument reported Cronbach’s *α* = 0.72, and the Korean version reported Cronbach’s α = 0.77. In the present study, Cronbach’s α was 0.81.

#### Menopause-specific quality of life

Menopause-specific quality of life was measured using the MENQOL-K developed by Hilditch et al. (6) and translated and validated in Korean by Park et al. (30). The instrument consists of 28 items rated on an 8-point Likert scale, with total scores ranging from 28 to 224. Higher scores indicate poorer menopause-specific quality of life. In line with other measures, scores were computed as mean item scores to enable interpretation within the original response scale and to enhance comparability across variables. The original instrument reported Cronbach’s *α* = 0.86, the Korean version 0.92, and in the present study, Cronbach’s α was 0.96.

Although the MENQOL consists of four domains (vasomotor, psychosocial, physical, and sexual), the total score was used in this study to represent overall menopause-specific quality of life. This approach was adopted because the primary aim of the study was to examine the overall effect of aging anxiety on quality of life and to test the mediating roles of psychological resources in an integrated manner, rather than focusing on domain-specific differences. Previous studies have also used the total MENQOL score as an indicator of overall health status and adaptation during menopause (2, 9).

### Statistical analysis

Statistical analyses were conducted using IBM SPSS Statistics version 26.0 (IBM Corp., Armonk, NY, United States). Descriptive statistics, including frequencies, percentages, means, and standard deviations, were calculated to summarize the general characteristics of the participants and the study variables. Pearson’s correlation coefficients were computed to examine the relationships among the main variables.

To examine the mediating effects of self-efficacy and self-acceptance on the relationship between aging anxiety and menopause-specific quality of life, Hayes’ PROCESS macro for SPSS (Model 4) was employed. Indirect effects were assessed using 5,000 bootstrap resamples, and a mediating effect was considered significant when the 95% confidence interval (CI) did not include zero. Statistical significance was set at *p* < 0.05.

Prior to conducting the analyses, statistical assumptions were assessed. Normality was evaluated using skewness and kurtosis values, and homogeneity of variance was assessed using Levene’s test.

## Results

### Participant characteristics

The general characteristics of the 114 participants are summarized in Table 1.

**Table 1:** Descriptive characteristics of study participants (*N* = 114).

Variable	Category	*n* (%)
Age	40–49	66 (57.9)
	50–59	48 (42.1)
Education	≤High school	55 (48.2)
	≥College	59 (51.8)
Marital status	Married	103 (90.4)
	Unmarried	4 (3.5)
	Other	7 (6.1)
Employment	Yes	75 (65.8)
	No	39 (34.2)
Monthly income	<4 million KRW	50 (43.9)
	≥4 million KRW	64 (56.1)
Number of children	≥1	107 (93.9)
	None	7 (6.1)
Menstrual status	Regular	63 (55.3)
	Irregular	22 (19.3)
	Menopause	29 (25.4)

Values are presented as *n* (%).

Participants aged 40–49 years accounted for 66 (57.9%), while those aged 50–59 years accounted for 48 (42.1%). Regarding education level, 59 participants (51.8%) had a college degree or higher, and 55 (48.2%) had a high school education or lower. Most participants were married (*n* = 103, 90.4%). Seventy-five participants (65.8%) were employed, whereas 39 (34.2%) were unemployed. In terms of monthly household income, 64 participants (56.1%) reported an income of ≥4 million KRW, and 50 (43.9%) reported <4 million KRW. Most participants had at least one child (*n* = 107, 93.9%), while 7 (6.1%) had no children. Regarding menstrual status, 63 participants (55.3%) reported regular menstruation, 29 (25.4%) were menopausal, and 22 (19.3%) reported irregular menstruation.

### Descriptive statistics of study variables

The means, standard deviations, and ranges of the major study variables are presented in Table 2. The reported means represent mean item scores rather than summed total scores; therefore, they are expressed within the original response scale range of each instrument. The mean score for aging anxiety was 2.78 ± 0.71 (range 1.2–4.8). Self-efficacy had a mean of 3.12 ± 0.57 (range 1.5–4.5), and self-acceptance had a mean of 3.45 ± 0.62 (range 2–5). The mean score for menopause-specific quality of life was 4.14 ± 1.02 (range 1–7).

**Table 2:** Descriptive statistics of study variables (*N* = 114).

Variable	Mean	SD	Min	Max
Aging anxiety	2.78	0.71	1.20	4.80
Self-efficacy	3.12	0.57	1.50	4.50
Self-acceptance	3.45	0.62	2.00	5.00
Menopause-specific quality of life	4.14	1.02	1.00	7.00

Values are presented as mean ± standard deviation. Scores represent mean item scores based on the original response scale of each instrument.

### Correlations among study variables

Pearson correlation analyses were conducted to examine the relationships among the major study variables. Aging anxiety was negatively correlated with self-efficacy (*r* = −0.37, *p* < 0.001) and self-acceptance (*r* = −0.50, *p* < 0.001), and positively correlated with menopause-specific quality of life (*r* = 0.50, *p* < 0.001). Self-efficacy was positively correlated with self-acceptance (*r* = 0.42, *p* < 0.001) and negatively correlated with menopause-specific quality of life (*r* = −0.40, *p* < 0.001). Self-acceptance was negatively correlated with menopause-specific quality of life (*r* = −0.50, *p* < 0.001).

### Parallel mediation analysis

A parallel mediation analysis was conducted using the PROCESS macro (Model 4) with 5,000 bootstrap resamples. The path coefficients for the model are presented in Table 3.

**Table 3:** Path coefficients for the parallel mediation model.

Path	B	SE	*t*	*p*	95% CI
Aging anxiety → Self-efficacy	−0.27	0.07	−3.76	<0.001	[−0.41, −0.13]
Aging anxiety → Self-acceptance	−0.27	0.04	−6.11	<0.001	[−0.36, −0.18]
Self-efficacy → MENQOL	−0.53	0.35	−1.53	0.128	[−1.21, 0.15]
Self-acceptance → MENQOL	−0.80	0.55	−1.46	0.148	[−1.90, 0.29]
Aging anxiety → MENQOL(c′)	1.40	0.30	4.74	<0.001	[0.81, 1.98]

MENQOL, menopause-specific quality of life. All coefficients are unstandardized (B).

Aging anxiety had a significant direct effect on menopause-specific quality of life (B = 1.40, SE = 0.30, t = 4.74, *p* < 0.001, 95% CI [0.81, 1.98]). Aging anxiety also had significant effects on self-efficacy (B = −0.27, SE = 0.07, t = −3.76, *p* < 0.001) and self-acceptance (B = −0.27, SE = 0.04, t = −6.11, *p* < 0.001). However, neither self-efficacy (B = −0.53, SE = 0.35, t = −1.53, *p* = 0.128) nor self-acceptance (B = −0.80, SE = 0.55, t = −1.46, *p* = 0.148) had a statistically significant effect on menopause-specific quality of life.

The indirect effects of aging anxiety on menopause-specific quality of life are presented in Table 4. The total indirect effect through self-efficacy and self-acceptance was statistically significant (B = 0.36, SE = 0.17, 95% CI [0.03, 0.71]). However, the specific indirect effects through self-efficacy (B = 0.14, SE = 0.11, 95% CI [−0.04, 0.42]) and self-acceptance (B = 0.22, SE = 0.16, 95% CI [−0.14, 0.50]) were not statistically significant, as their confidence intervals included zero.

**Table 4:** Indirect effects of aging anxiety on menopause-specific quality of life.

Effect	B	SE	95% CI
Total indirect effect	0.36	0.17	[0.03, 0.71]
Self-efficacy	0.14	0.11	[−0.04, 0.42]
Self-acceptance	0.22	0.16	[−0.14, 0.50]

Indirect effects were estimated using 5,000 bootstrap samples. Confidence intervals are 95% bias-corrected. All coefficients are unstandardized (B).

The overall model explained 33.3% of the variance in menopause-specific quality of life (*R*^2^ = 0.33).

**Figure 1:**
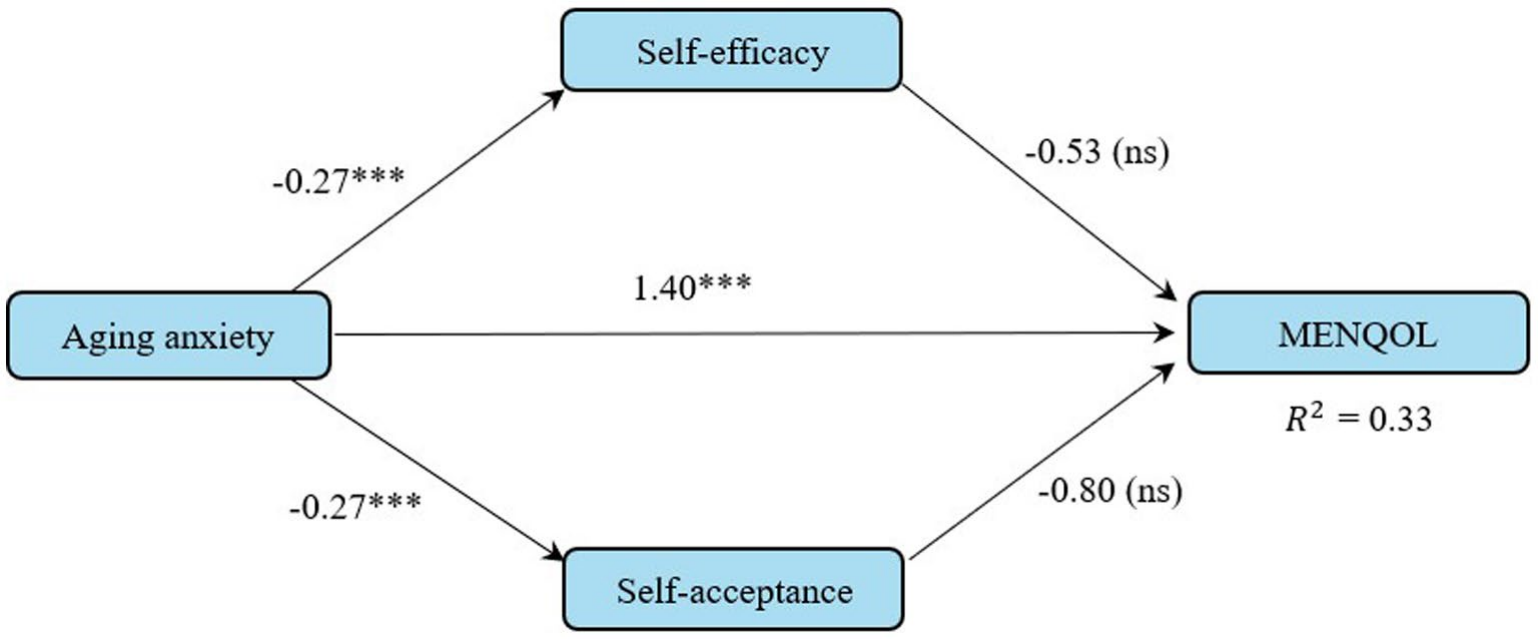
Parallel mediation model. MENQOL, menopause-specific quality of life. All coefficients are unstandardized (B). *** *p* < 0.001; ns = not significant.

The final parallel mediation model, including the unstandardized path coefficients, is illustrated in Figure 1.

## Discussion

This study examined the effects of aging anxiety on menopause-specific quality of life and investigated the parallel mediating roles of self-efficacy and self-acceptance among women experiencing menopausal symptoms. The findings indicate that aging anxiety had a significant direct effect on quality of life, and the total indirect effect through self-efficacy and self-acceptance was significant, although the specific indirect effects of each mediator were not statistically significant, suggesting that these psychological resources may operate in a combined manner as parallel mediators. These findings suggest that menopause-specific quality of life may be explained by complex psychological processes rather than a single factor.

### Main findings and interpretation

Consistent with previous studies, higher levels of aging anxiety were associated with higher MENQOL scores, indicating poorer menopause-specific quality of life (10). Higher levels of aging anxiety have been associated with decreased quality of life, and the present findings further support this relationship (10). This may be explained by the fact that negative perceptions of aging can increase emotional distress and psychological stress, which may heighten sensitivity to physical symptoms and reduce overall life satisfaction (10, 13). Moreover, women experiencing menopausal symptoms may experience both biological and psychosocial changes simultaneously, which may be associated with a stronger relationship between aging anxiety and quality of life (4, 21).

Although self-efficacy did not demonstrate a statistically significant individual mediating effect, it may function as a psychological resource in the relationship between aging anxiety and quality of life. These findings are consistent with Social Cognitive Theory (15). According to this theory, self-efficacy is defined as an individual’s belief in their capability to perform the actions required in specific situations, and this belief influences emotional regulation, stress coping, and goal-directed behaviors. In particular, when aging-related negative perceptions, such as aging anxiety, induce emotional distress and reductions in quality of life, self-efficacy can help individuals exert control over the situation and adapt effectively (14, 16). In other words, individuals with higher self-efficacy are more likely to respond effectively to stress arising from negative emotions or physical changes, maintain health-promoting behaviors, and partially mitigate declines in quality of life, thereby functioning as a psychological resource (14, 16).

Similarly, self-acceptance did not demonstrate a statistically significant individual mediating effect, but it may still play a meaningful psychological role. These findings are consistent with Ryff’s (19, 20) Psychological Well-Being (PWB) theory. According to Ryff, psychological well-being is a multidimensional construct reflecting an individual’s overall evaluation of life, comprising six key dimensions: self-acceptance, purpose in life, autonomy, environmental mastery, personal growth, and positive relations with others. Among these, self-acceptance refers to the capacity to acknowledge and accept both positive and negative aspects of oneself, to positively evaluate past experiences and the current self, and it plays a central role in emotional stability and life satisfaction.

In the present study, self-acceptance in women experiencing menopausal symptoms likely contributed to mitigating the negative impact of aging anxiety on quality of life by enabling them to positively accept their physical changes and psychological state while maintaining emotional balance. Although the individual indirect effects were not statistically significant, self-acceptance aligns with the psychological functions described in Ryff’s (19, 20) self-acceptance dimension and may be considered a psychological resource that could contribute to the maintenance of quality of life and emotional adaptation in menopausal women. This interpretation is also supported by Yoshitake et al. (31), which reported that self-acceptance may play an important role in interpreting and reconstructing the meaning of life.

In the present study, while neither self-efficacy nor self-acceptance demonstrated statistically significant individual mediating effects, the total indirect effect considering both variables simultaneously was statistically significant. This supports the interpretation that, based on Bandura’s Social Cognitive Theory (15) and Ryff’s Psychological Well-Being Theory (19, 20), self-efficacy and self-acceptance may jointly function as psychological resources in the relationship between aging anxiety and quality of life.

In this context, recent studies (2023–2025) have reported both consistent and inconsistent findings regarding the relationships between psychological factors and menopause-specific quality of life, suggesting that these associations may vary depending on sample characteristics, cultural context, and methodological approaches.

Although direct comparison with previous studies is limited due to the scarcity of relevant literature, the present findings show some differences when compared with prior research. In the study by Magistro et al. (32), self-efficacy was found to have a statistically significant partial mediation effect on quality of life. In contrast, Yoshitake et al. (31) reported that self-acceptance may play an important role in interpreting and reconstructing the meaning of life.

These results suggest a potential role for additional psychological factors and contextual influences in explaining why the individual indirect effects were not statistically significant in the present study (24). For example, other psychological or environmental factors—such as conflict resolution skills, self-identity, stress coping abilities, social support, and emotion regulation—may have partially influenced the outcomes (22, 31). The involvement of these factors may have contributed to the non-significance of the individual indirect effects of self-efficacy and self-acceptance, indicating that quality of life is influenced not only by single psychological variables but also by a complex interplay of social and environmental factors.

These findings suggest that a portion of the relationship between aging anxiety and quality of life remains unexplained by the mediators included in this study, indicating the potential involvement of additional psychological or contextual factors.

These findings suggest that psychological factors may be more appropriately considered in combination rather than individually. While previous studies have primarily focused on individual psychological factors (2, 3), the present study provides evidence suggesting that multiple psychological resources may operate together, contributing to a more comprehensive understanding of the psychological processes underlying quality of life in women experiencing menopausal symptoms.

Although the total indirect effect was statistically significant, its magnitude was relatively small. Considering the small effect size, the limited sample size, and the fact that participants were drawn from a specific region, the practical relevance of these mediation effects should be interpreted with caution, and statistical significance alone should not be overgeneralized.

### Implications

The findings of this study have important implications for nursing practice and health promotion programs aimed at improving the quality of life of women experiencing menopausal symptoms. Because quality of life is associated with the interaction of multiple psychological factors, it is important to consider psychological resources alongside the management of physical symptoms (22).

Accordingly, intervention strategies may extend beyond the management of physical symptoms to include approaches that support both self-efficacy and self-acceptance. For example, combining cognitive and behavioral interventions designed to strengthen self-efficacy with psychological counseling and emotional support programs to promote self-acceptance may allow these psychological resources to work together and contribute to improvements in quality of life (17, 31). These findings suggest the need for integrated and multidimensional intervention approaches.

However, considering that the magnitude of the indirect effects observed in this study was relatively small, the sample size was limited, and the participants were drawn from a specific region, caution is warranted when applying these findings to practice. The results should not be overgeneralized, and their practical or clinical relevance should be interpreted carefully within the context of these limitations.

### Limitations and future directions

This study has several limitations. First, as a cross-sectional study using secondary data, causal relationships among variables cannot be clearly established. Second, the use of self-reported questionnaires may have introduced response bias. Third, the study population was defined as women experiencing menopausal symptoms rather than all menopausal women, and the sample was recruited using a convenience sampling method, which may restrict the generalizability of the findings.

In addition, although MENQOL is a multidimensional instrument consisting of four domains, this study used the total score, for the purpose of simplified and integrated analysis, which may not fully reflect differences across individual domains. Therefore, caution is needed when interpreting the findings, as domain-specific characteristics of menopause-specific quality of life may not have been fully captured.

In addition, the sample size in this study was determined based on multiple regression analysis and may not have been sufficient to detect individual indirect effects in the mediation model. Previous studies have suggested that larger sample sizes are required to achieve adequate statistical power for mediation analysis, particularly when effect sizes are small or moderate (24, 25, 33). Therefore, the non-significant individual indirect effects observed in this study should be interpreted with caution. In addition, although a widely used and validated instrument was employed to measure self-efficacy, it is based on an earlier adaptation of the original scale, and future studies may benefit from incorporating more recently developed measures.

Furthermore, this study did not include key sociodemographic covariates such as age, income, and marital status in the regression model. The absence of these control variables may allow confounding effects to influence the relationships between psychological factors and menopause-specific quality of life, potentially leading to over- or underestimation of regression coefficients. Therefore, the findings should be interpreted with caution. Future research is recommended to incorporate these sociodemographic variables to enhance the reliability and validity of the results.

Future studies should include more diverse populations across different regions and cultural contexts and employ longitudinal designs to better examine the relationship between aging anxiety and quality of life. In addition, further research is needed to explore extended mediation models that include a broader range of psychological factors, particularly to further clarify how multiple psychological resources operate in combination. Intervention studies may also be useful to evaluate the effectiveness of programs designed to simultaneously enhance self-efficacy and self-acceptance.

## Conclusion

This study examined the parallel mediating roles of self-efficacy and self-acceptance in the relationship between aging anxiety and menopause-specific quality of life among women experiencing menopausal symptoms. The findings suggest that aging anxiety had a significant direct effect on menopause-specific quality of life, and the total indirect effect through self-efficacy and self-acceptance was significant, although the individual indirect effects of each mediator were not statistically significant. Self-efficacy and self-acceptance may operate jointly rather than independently as mediators in this relationship.

These findings suggest that interventions aimed at improving the quality of life of women experiencing menopausal symptoms may benefit from strategies that reduce aging anxiety and enhance self-efficacy and self-acceptance. Specifically, programs focusing on goal-setting and behavioral planning to strengthen self-efficacy, as well as cognitive restructuring approaches to promote self-acceptance, may be useful as practical strategies.

This study has several limitations. The use of cross-sectional secondary data limits causal inference, and the sample was drawn from a specific region, which may restrict generalizability. Future research should include more diverse populations across different regions and cultural contexts and employ longitudinal designs to better examine the relationships among aging anxiety, psychological resources, and quality of life.

## Data Availability

This study used previously collected secondary data. All data were anonymized to ensure participant confidentiality, and access is restricted to the research team for the purposes of this study only. Requests to access the dataset can be made by contacting the corresponding author, JK, via email: jung88922@naver.com.
